# Public communication can facilitate low-risk coordination under surveillance

**DOI:** 10.1038/s41598-022-07165-9

**Published:** 2022-03-02

**Authors:** Amos Korman, Pierluigi Crescenzi

**Affiliations:** 1 French-Israeli Laboratory on Foundations on Computer Science, UMI FILOFOCS, CNRS, UP7, TAU, HUJI, WIS, International Joint Research Unit, Tel-Aviv, Israel; 2 Gran Sasso Science Institute, 67100 L’Aquila, Italy

**Keywords:** Computational science, Computer science, Statistics, Stochastic modelling, Computational models

## Abstract

Consider a sub-population of rebels aiming at initiating a revolution. To avoid initializing a failed revolution, rebels would first strive to estimate their “power”, which is often correlated with their number. However, especially in non-democratic countries, rebels avoid disclosing themselves. This poses a significant challenge for rebels: estimating their number while minimizing the risk of being identified as rebels. This paper introduces a distributed computing framework to study this question. Our main insight is that the communication pattern plays a crucial role in achieving such a task. Specifically, we distinguish between *public communication*, in which each message announced by an individual can be viewed by all its neighbors, and *private communication*, in which each message is received by one neighbor. We describe a simple protocol in the public communication model that allows rebels to estimate their number while keeping a negligible risk of being identified as rebels. The proposed protocol, inspired by historical events, can be executed covertly even under extreme conditions of surveillance. Conversely, we show that under private communication, protocols of similar simplicity are either inefficient or non-covert. These results suggest that public communication can facilitate the emergence of revolutions in non-democratic countries.

## Introduction

Large scale changeovers in a population, such as political overthrows of dictatorships by rebels, are often perceived as complex emergent phenomena^1–4^. In order to avoid conducting a failed rebellion, rebels would typically refrain from initiating a revolution until they manage to obtain a reliable indication that many others will join them^5,6^. In other words, rebels would first try to understand whether “*we* are the *many* and *they* are the *few*”^7^. However, and especially under non-democratic regimes, obtaining such information may not be a trivial task, as rebels often refrain from disclosing themselves as such. In turn, with the lack of such information, the status quo may continue to hold even when the support for a revolution is effectively high. Nevertheless, in some cases, rebels successfully coordinate their actions even under severe conditions of surveillance. It is currently not well understood which conditions are crucial for coordination between rebels and, conversely, under which circumstances coordination is bound to fail. Progress in this direction may be valuable towards better understanding the constraints that govern the emergence of revolutions.

In this paper we take a general stance to identify basic conditions that could facilitate coordination between rebels despite facing extreme forms of surveillance. Drawing on reasoning from the field of distributed computing^8^, we argue that the pattern of communication can play a key role in the ability of rebels to reach such a coordination safely. To characterize our results, we distinguish between two extreme patterns in a network environment:

- *Private communication* Each message sent by a participant is heard by a single neighbor.
- *Public communication* Each message sent by a participant is heard by all its neighbors.

The distinction between public and private communication is common in the distributed computing and computer network disciplines. Depending on the context, the former type of communication is often referred to as “broadcast”, while the latter is referred to as “unicast”^8,9^. Private communication aims to capture one-to-one interactions, which are executed either directly in person or via a digital private messaging platform. In contrast, public communication aims to model social media infrastructures, such as Facebook or Twitter, that are believed to have played a significant role during recent revolutions, including the Arab spring, Ethiopian, and the Lebanese revolutions^2,10,11^. In addition, public communication can also capture behavioral communication, such as the “slow-motion” day organized by the opponents of the Pinochet dictatorship in the 1970s^6^, crowd assemblies, such as the one gathered during the last public speech by Ceauescu in 1989^3^ (see below for more details), or even chemical communication during quorum-sensing by pathogenic bacteria before attacking their host^12^ (see “Discussion and future works” for more details regarding quorum-sensing by bacteria.)

The parallel nature of public communication can allow for fast information spread. This is commonly considered a fundamental property that explains how the use of social media helped accelerate several social movements^2^. Here we argue that from the perspective of rebels, there is another significant benefit of public communication: It not only facilitates fast information spread but also allows for its covert dissemination. More specifically, we claim that as long as the typical degree in the network is not too small, public communication can facilitate the ability of agents belonging to a sub-population to estimate their proportion in the population while keeping a negligible risk of each rebel being identified as such. Conversely, we show that under private communication, protocols of similar simplicity are either inefficient or non-covert.

In light of these insights, it may be interesting to revisit the emergence of past revolutions, especially in non-democratic countries. For example, a pivotal moment in the Romanian revolution was the botched public speech that Ceauescu gave on 21 December 1989. In the wake of growing social tension, Ceauescu conducted a speech before a dense crowd consisting of tens of thousands in Palace Square. Aiming to demonstrate the control of the leader, the speech was nationally televised to millions. The crowd was given orders on when to applaud and what to chant, while secrete policemen were scattered among the crowd making sure that everything is in order. Such a speech was conducted yearly, but this time something different happened. In the beginning of the speech, the crowd stayed quite when Ceauescu spoke, applauded the leader at intermediate pauses, and chanted admiration songs. However, eight minutes into the speech, some sound began to arise from the crowd which became louder and louder until the crowd started booing. Ceauescu and his wife Elena fled the scene by helicopter; A day after they were captured, put on trial, and shot by a firing squad. It is unclear what dynamics led to the dramatic switch in the crowd’s behavior, from being completely submissive to rebellious. Among other factors, it appears plausible that despite the surveillance, rebels in the crowd managed to somehow understand that if they suddenly act rebelliously, sufficiently many others would join. Our results suggest that the high density of the assembly could have had a non-negligible contribution to the emergent changeover in the crowd’s behavior (see more details in “Discussion and future works”).

To obtain some intuition regarding the difficulties involved in the rebels estimating their proportion in the population without revealing that they are rebels, let us consider a simple setting with two communicating participants: Alice and Bob. A possible scenario can be the following. Being a rebel, Alice would try to understand whether Bob is also a rebel. While talking normally, she could start by “cautiously tempting” Bob into sending “rebellious” signals. If Bob would be a rebel, then, in turn, he may “cautiously respond” to Alice by sending some, but not too many, rebellious signals, and, in parallel, try to “cautiously tempt” Alice into doing the same. At the end of the conversation, each person would classify the other as a rebel if the (weighted) number of rebellious signals he or she received passes a certain threshold. Unfortunately, in such a scenario, unless employing some sophisticated cryptographic protocol (which is highly unlikely in direct communication between humans), there is little hope for rebels if the police are surveilling all conversations. Indeed, if both rebels could detect that the other person is a rebel, e.g., by counting the weighted rebellious signals he or she sends, then so could the police. In fact, the same argument holds with respect to any evaluation criterion used by one of the parties that takes as input only the conversation between the parties (and not, for example, some random private key generated by a party before the execution starts as could be done using cryptographic techniques^13^). However, these difficulties do not rule out the possibility that simple covert estimation mechanisms exist in a multi-party scenario. Indeed, in contrast to the two-party scenario, understanding that there are many rebels in a large population does not necessarily imply that one can identify who they are.

### Related works

This paper studies the problem of distributively estimating the relative size of a sub-population whose members refrain from disclosing themselves as such. Unlike multiple works in the distributed computing and computer network disciplines that study size estimation^14–20^, our work initiates the analytical study of such procedures in the presence of surveillance, which imposes direct risk on individuals in such a sub-population that identify themselves as such.

Secure computation under cryptographic assumptions was introduced by Yao^13^. By now, there is a huge body of literature on secure computations, in both two-party scenarios and multi-party scenarios, including the case of tolerating a quorum of Byzantine players^21^, and making known distributed algorithms secure^22^. The concept of covert computation was introduced in^23^ for two-party scenarios and in^24^ for multi-party scenarios. The idea behind covert protocols is that parties do not know if other parties are also participating in the protocol or not. In general, however, most of schemes in the cryptography literature employ sophisticated operations on both the encoding and the decoding parts (e.g., many of the protocols are based on first generating a huge random number and then manipulating it in a sophisticated manner^13^). While such operations can be implemented by computers, they cannot be expected to be employed directly by humans or other biological entities.

Here, we are mostly interested in simple estimation protocols, based on sampling and sensing a certain tendency. Such mechanisms are natural for humans and other biological entities^25^. For example, similar protocols are executed by bacteria communities aiming to identify when their density passes a certain threshold^26^.

### Model

This paper introduces a distributed computing framework that aims to study covert population-size estimation by humans and other biological entities. For this purpose, we give special attention to simplicity, in both message encoding, and decoding. In particular, we assume that messages are real numbers that, in the context of revolution, represent a certain level of satisfaction with the ruling entity. In turn, the decoding is assumed to be threshold-based, capturing the ability to sense a certain tendency.

Formally, we consider an idealized model consisting of *n* *agents*, communicating over a network *G*, where the nodes represent the agents and the edges represent communication links between neighbors. The *degree* of an agent *i*, denoted $\Delta _i$, is the number of neighbors of *i* in *G*. Let $\Delta$ denote the median degree.

Initially, each agent is chosen as a *rebel* with probability $0\le \rho \le 1$, independently of others, and otherwise, it is an *obedient citizen*. The independence assumption abstracts away correlations in behavior between nearby agents, and is made for simplification of analysis. A priori, the behavior type of an agent is known to itself but not to others. The parameter $\rho$, which can be considered as the fraction of rebels in the population, is unknown to the agents. We say that there are “many rebels” if $\rho \ge 0.8$, and “few rebels” if $\rho \le 0.2$, where it should be clear that the constants 0.8 and 0.2 were chosen arbitrarily and any other constants $1>c_1>c_2>0$ could have been used instead (while changing other constants in the definitions below accordingly). Informally, the aim of the rebels is to distinguish the case of many rebels from the case of a few rebels while minimizing the risk of disclosing themselves as rebels.

As mentioned, we distinguish between public and private communication models. In either case, we assume that communication consists of a single round, in which agents exchange messages in parallel, so that each agent has access to (a distorted version of) a message sent by each of its neighbors. Hence, each agent *i* sends $\Delta _i$ messages and receives the same amount of messages. The restriction to one round is made for the sake of simplicity of definitions, however, it should be clear that our framework can be extended to multiple rounds.

Each message is modeled as a real number, which may encode information about the level of satisfaction with the ruling entity. For simplicity and normalization purposes, we assume that the messages sent by obedient citizens are always equal to 0. The value 0 aims to represent the standard or neutral level of satisfaction with the ruling entity that is implicitly communicated in a typical message sent by a non-rebel. In contrast, rebels execute a specified *rebel protocol*, which can allow them to freely choose the messages they send. For example, a rebel may send a message that is close to 0, thus enabling it to pretend to be an obedient citizen, or send a very large message, to clearly signal other that it is a rebel.

Unless mentioned otherwise, we consider only deterministic rebel protocols. Moreover, when considering the private communication model, we restrict attention to *uniform* rebel protocols, in which the same message is sent to all neighbors. Such protocols are natural analogs of protocols in the public communication model. Hence, in both private and public models, a rebel deterministically decides on a single message *m* to be delivered to its neighbors. The difference between the two models is that, in the private model, an agent *i* actively sends $\Delta _i$ copies of *m* (one copy per neighbor), whereas, in the public model, it only announces the message *m* once, and then this message is heard by all its neighbors.

Importantly, in both models, the receiver of a message may not interpret the corresponding information correctly. To capture this, we assume that every message $m_{i,j}$, originated at agent *i* and heard by agent *j*, is received by *j* as

$$\begin{aligned} s_{i,j} = m_{i,j} +N(0,1), \end{aligned}$$

where *N*(0, 1) is a normally distributed variable. This noise variable is sampled for each neighbor *j* of agent *i*, independently from all other neighbors of *i*. In order to avoid confusion between a message $m_{i,j}$ and its “distorted” version $s_{i,j}$, we refer to the latter as a *signal*.

All rebels execute the same rebel protocol (when considering randomized protocols, the execution of a rebel may also depend on the results of random coins tossed by the agent). For deterministic protocols, all probability measures defined below are made with respect to the distribution of rebels in the population, and the noises in the messages. (For randomized algorithms, probability measures are defined also with respect to the coin tosses of agents).

At the end of the communication round, after receiving the signals from all its neighbors, each rebel either outputs “many”, or does not output anything. The *success probability* of a rebel protocol, denoted $p_{\text {success}}$, is the probability that at least a third of the rebels output “many” when $\rho \ge 0.8$ (as before, the constant 1/3 is arbitrary). The *output-risk* of a rebel, denoted $r_{\text {output}}$, is defined as the probability that it outputs “many”, given that $\rho \le 0.2$. This captures the risk of having a false positive.

Another component of the risk corresponds to being identified as a rebel as a result of sending too suspicious messages. This risk depends not only on the messages sent by a rebel but also on the abilities of the surveilling entity, called *police*, and on the criteria it uses to identify rebels. Aiming to capture extreme conditions associated with totalitarian countries, we assume that the police surveils all communication links. However, for fairness considerations, similarly to the agents in the system, the police cannot see the actual messages sent and instead sees their corresponding signals. That is, every message *m* sent by an agent is seen by the police as a signal $s = m +N(0,1)$, where the sample noise is independent of all other events. For each agent *i*, the police protocol considers the signals associated with all the messages *i* sends and then decides whether or not to *arrest* the agent. Note that under public communication, the police receives one signal from each agent *i* (since agent *i* announces one message only), whereas in the private communication model it receives $\Delta _i$ such signals.

The police’s goal is to arrest as many rebels as possible while minimizing arrests of obedient citizens. Being permissive with respect to the police, we assume that it’s computational power is unlimited, and that it knows both the rebel protocol and the fraction of rebels $\rho$. Conversely, being restrictive with respect to rebels, we assume that rebels do not know the police’s protocol, and must guarantee low risk with respect to any police protocol. Specifically, the *relative message-risk* of a rebel protocol, denoted $r_{\text {message}}$, is defined as the maximal difference between the probability that a rebel is arrested by the police and the probability that an obedient citizen is arrested, where the maximum is taken over all police protocols. If the police protocol is randomized, then the probability $r_{\text {message}}$ is calculated also with respect to the randomness in the police protocol (in addition to the aforementioned sources of randomness). Finally, the *total risk* of a rebel is the output-risk plus the relative message-risk:

$$\begin{aligned}r_{\text {total}}=r_{\text {output}}+r_{\text {message}}.\end{aligned}$$

To illustrate the definitions, let us briefly discuss two trivial protocols operating under the public communication model. In the first protocol each rebel outputs “many” regardless of the messages it receives. There, the success probability is extremely high, namely 1, but so is the output-risk. A second trivial protocol imitates the behavior of obedient citizens by letting each rebel announce the message zero. This protocol has relative message-risk that is equal to zero, but regardless of its outputting rule (concerning when to output “many”), it cannot maintain both high success probability and low output-risk.

In summary, the goal of the rebels is to (1) maximize the success probability, and at the same time (2) minimize the total risk.

## Results

This paper introduces a distributed computing framework that aims to study covert computations by humans or other biological entities. Our focus is on the ability of a sub-population of agents (rebels) to perform very simple computations to estimate their fraction in the population, while minimizing the risk of exposing the fact that they belong to the sub-population. The model assumes that other agents (obedient citizens) simply send the number zero to their neighbors, which in turn receive a distorted version of this number due to noise. Our main takeaway message is that even under extreme surveillance conditions, there are simple deterministic protocols in the public communication model that allow rebels to estimate their fraction in the population while keeping a negligible risk of each rebel being identified as such. Conversely, we show that under a peer-to-peer analogue, protocols of similar simplicity are either inefficient or non-covert. We next describe our results in more details.

### The *Quorum-Sensing* protocol

**Figure 1:**
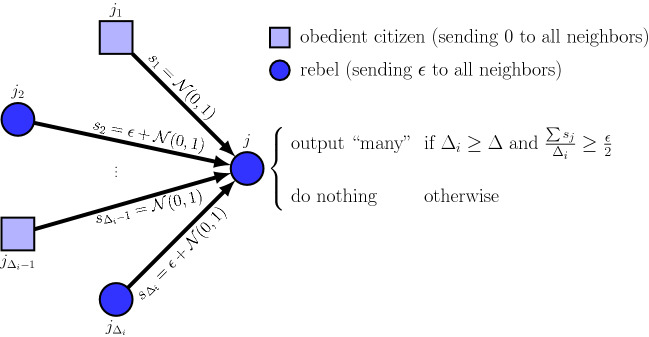
The Quorum-Sensing protocol with parameter $\varepsilon$. Rebels send the number $\varepsilon >0$ to all their neighbors, and obedient citizens send the number 0. Rebel *i* receives signals from its $\Delta _i$ neighbors (a combination of rebels and obedient citizens), and decides whether to output “many” according to the rule specified on the right ($\Delta$ denotes the median degree).

We first consider an extremely simple rebel protocol, termed *Quorum-Sensing*, which is particularly useful when executed in the public communication model (see Fig. 1). Adapted to the context of revolution, the idea may be considered as a variant of the randomised response method used in survey interviews to allow respondents answer sensitive issues while preserving confidentiality^27^. The Quorum-Sensing protocol is also inspired by historical events that happened during the Pinochet dictatorship in the 1970s. The rebels opposing Pinochet used the idea of suggesting to act slowly, for example, that taxi drivers would drive slower than usual. The message spread rapidly, and many people cooperated in this initiative^6^. Watching the city’s low motion, the rebels could realize that they were many without incurring considerable risk.

Formally, in this protocol, each rebel simply sends the message $m=\varepsilon$, for some predetermined parameter $\varepsilon >0$. At the end of the communication round, a rebel outputs “many” if and only if (1) its degree is at least the median degree $\Delta$, and (2) the average value of a signal received from a neighbor is at least $\varepsilon /2$.

The theorem below states that under the public communication model, $\varepsilon$ can be set to be sufficiently small to guarantee that the total risk incurred by this protocol is arbitrarily small, while still maintaining extremely high success probability on highly connected networks.

#### Theorem 1

*Consider the public communication model and a network with median degree* $\Delta$. *Consider a fixed* $\varepsilon >0$. *For any sufficiently large* *n* and $\Delta >c\log n$ *for a sufficiently large constant* *c, the success probability of the Quorum-Sensing protocol is at least* $1-O(1/n^2)$, *while the total risk is at most* $0.715\varepsilon$.

The formal proof of the theorem is given in the SI, Sect. C. Intuitively, the reasoning behind the proof is as follows. Under public communication, each agent sends only one message. If this message is sufficiently close to what an obedient citizen sends, i.e., close to 0, then the rebel can hide behind the noise (see Lemma 1). On the other hand, when observing the signals coming from many agents, a small bias in the original messages of many rebels becomes visible, due to the law of large numbers that effectively cancels noise.

**Figure 2:**
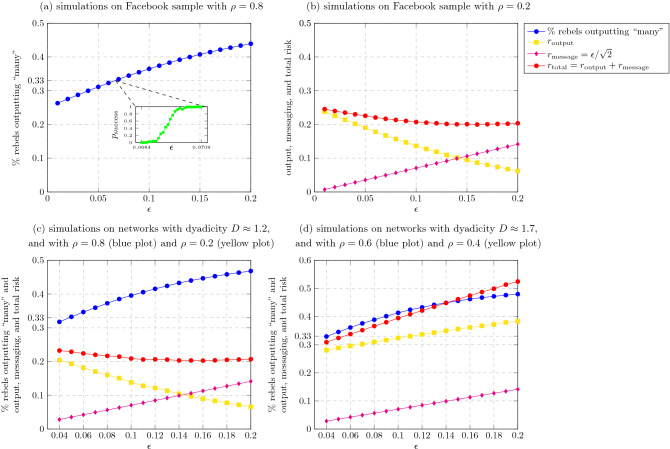
Public communication: Simulations of the Quorum-Sensing protocol on a Facebook network. We ran 10 times the Quorum-Sensing protocol on the sample of the Facebook social graph released in^28,29^ (see “Methods”). The (average) percentage of rebels which output “many” when $\rho =0.8$ (i.e, “many rebels”) is shown in (**a**). The inset in (**a**) represents the success probability in the interval between 0.0684 and 0.0709, that is, the probability that the percentage of rebels which output “many” is greater than $\frac{1}{3}$. The (average) risks of a rebel are depicted in (**b**). The $r_\text {output}$ value denotes the percentage of rebels which output “many” when $\rho =0.2$ (i.e, “few rebels”), while $r_\text {message} = \varepsilon /\sqrt{2}$; this latter value is, indeed, an upper bound on the message risk, as shown in Lemma 1. Finally, $r_\text {total}$ is simply the sum of $r_\text {output}$ and $r_\text {message}$ (because of the previous observation, this is an upper bound on the total risk). (**c**) and (**d**) correspond to simulations on networks with different levels of homophily. Specifically, (**c**) corresponds to networks with dyadicity $D\approx 1.2$ and (**d**) corresponds to networks with dyadicity $D\approx 1.7$. In (**c**), as usual, $\rho =0.8$ represents the case of “many rebels” while $\rho =0.2$ represents the case of “few rebels”. In (**d**), however, these values are replaced by 0.6 (for “many rebels”) and 0.4 (for “few rebels”); the reason for this exchange is explained in “Methods”. The semantics of the plots in (**c**) and (**d**) remain the same as in (**a**) and (**b**).

We corroborated this analytical result by conducting simulations of the Quorum-Sensing protocol over a real-world social network. The simulations were conducted on the Facebook graph released by Gjoka et al. in^28,29^, which was collected in April 2009, containing a sample of approximately 1.2 million users reached by one breadth-first-search traversal. See more details in “Methods”. The results are shown in Fig. 2a,b. From these two plots, we can conclude that the minimum value of the total risk $r_\text {total}$ is approximately 0.2 and it is obtained at roughly $\varepsilon =0.16$, which corresponds to a success probability of 1. Indeed, as it can be derived from the inset of the plot in Fig. 2a, the success probability is 1 for any $\varepsilon >0.0709$.

These results were obtained by assigning the rebel labels randomly at each node with probability $\rho$, independently of other nodes. The independence assumption is used in our model in order to simplify the analysis. In real-life scenarios, however, people of a “similar type” often tend to cluster. In our context, this phenomenon, commonly referred to as *homophily*^30^, implies that rebel nodes would be more frequently connected to other rebel nodes than to citizen nodes. Clearly, the higher the level of homophily is, the harder it is to infer the global fraction of rebels based on the set of neighbors only. In order to study the robustness of the Quorum-Sensing protocol to homophily, we conducted simulations over a subgraph of the Facebook network, by assigning rebel labels in a way the yields different levels of homophily, while maintaining that the fraction of rebels in the population remains $\rho$.

More precisely, in order to measure the degree of homophily of the labeled network we use the *dyadicity* measure *D* proposed in^31^. This measure equals to the ratio between the number of rebel/rebel edges and the expected number of such edges if the edges of the network were created randomly in an independent manner (see more details in “Methods”). If $D>1$, then the network is *dyadic* (when the assignment of rebel labels is independent between nodes, as we did in the simulations of Fig. 2a,b, $D\approx 1$). The results of the application of the Quorum-Sensing protocol to dyadic networks are shown in Fig. 2c,d. As it can be seen in Fig. 2c, when the dyadicity is $D\approx 1.2$ the results are similar to the case in which the rebel labels are assigned to the nodes independently (that is, without homophily). Indeed, the minimum value of the total risk $r_\text {total}$ is approximately 0.2 and is obtained at roughly $\varepsilon =0.16$, which corresponds to a success probability of 1. This suggests that the Quorum-Sensing protocol is robust with respect to a relatively low amount of homophily present in the network. On the other hand, as it can be seen in Fig. 2d, when the dyadicity is $D\approx 1.7$, the total risk $r_\text {total}$ significantly increases with the value of the parameter $\varepsilon$, thus making the Quorum-Sensing protocol less safe. In particular, the minimum value of the total risk $r_\text {total}$ corresponding to a success probability of 1 is approximately 0.32, that is, about $50\%$ higher than in the case of $D\approx 1.2$. (Note that the simulations corresponding to Fig. 2d have been produced with respect to a different interpretation of “many rebels” and “few rebels”, see “Methods” for more details).

In addition, we conducted simulations of the Quorum-Sensing protocol over “synthetic” networks obtained by applying the Chung-Lu model^32^ over the degree distribution of a somewhat more recent sample of the Facebook social graph, studied in^33^. These simulations are discussed in the SI, Sect. A. As it can be seen by comparing Fig. S6a to Fig. 2a,b, the results are quite similar to the results obtained in the case of the sample of the 2009 Facebook social graph. Moreover, in Sect. A we also conducted simulations of (a slightly modified version of) the Quorum-Sensing protocol over the same “synthetic” networks, but this time assuming that nodes have varying “strengths”. Intuitively, a “stronger” node is interpreted as having a larger impact on the success of the revolution, and we aim to see whether the accumulation of strengths of rebels who output “many” passes a certain threshold (see more details in SI, Sect. A). In these simulations, we focused on one particular assignment of strengths, in which the strength of a node equals to its degree in the graph. As it can be seen from Fig. S6, the results of these simulations are quite similar to the unweighted case, where all nodes have the same strength.

### Robustness to undercover agents

In some scenarios, *undercover agents* may secretly cooperate with the police. In the context of our model, such agents aim to distort the detection protocol of the rebels to reduce the rebels’ success probability or to increase the rebel’s output-risk. For example, the Quorum-Sensing protocol’s correctness is very sensitive to undercover agents. For instance, in the case of a complete network, even a single undercover agent can diminish the correctness of the Quorum-Sensing protocol by sending a message consisting of a huge number.

We next present a variant of the Quorum-Sensing protocol, called *Median*, which is robust to a non-negligible fraction of undercover agents. The protocol uses the same messaging protocol as the Quorum-Sensing protocol, i.e., it deterministically sends the message $\varepsilon$. Moreover, similarly to the Quorum-Sensing protocol, a rebel *i* outputs nothing if its degree is small, i.e., if $\Delta _i<\Delta$. Otherwise, it counts the number of incoming signals that are above $\varepsilon$, and outputs “many” if and only if the number of such signals exceeds $(\frac{1}{2}-\frac{7\varepsilon }{30})\Delta _i$.

The next theorem quantifies the robustness of the Median protocol to the presence of undercover agents. It suggests that for a range of relatively small $\varepsilon$, the Median protocol yields similar guarantees as the Quorum-Sensing protocol, even when facing a small fraction of undercover agents. The proof of the theorem is given in the SI, Sect. D.

#### Theorem 2

*Assume that the probability that an agent is undercover is* *o (1). If* $\varepsilon \in [0.04,0.2]$, *then there exists a constant* $c>0$ *such that for any sufficiently large* *n, and degree* $\Delta > \frac{c\log n}{\varepsilon ^2}$, *the success probability of the Median protocol is at least* $1-O(1/n^2)$, *and the total risk is at most* $0.715\varepsilon$.

The “o” notation in the theorem is with respect to the median degree $\Delta$. We further note that the particular constants we are going to use here are not meant to be optimized. Instead, these constants are used for convenience, as they are based on specific bounds on the tail distribution of a normal distribution.

### An impossibility result under private communication

In the context of private communication, each rebel executing the Quorum-Sensing protocol (or the Median protocol) would send the number $\varepsilon >0$ to each neighbor. Hence, instead of sending just one message $\varepsilon$ as in the public communication case, a rebel *i* now sends $\Delta _i$ such messages. The next theorem states that any such uniform deterministic protocol fails to provide both low total risk and high success probability, regardless of the decision of when to output “many”. The proof of the theorem appears in the SI, Sect. E.

#### Theorem 3

*Consider a private communication framework in a regular network of degree* $\Delta$ *of size n. Consider any uniform deterministic rebel protocol. Assume that the success probability is bounded away from zero for sufficiently large n* *and* $\Delta$, *that is, the success probability is at least p, for some constant* $p>0$. *Then the total risk of a rebel is at least* $\frac{p}{4}-\frac{1}{n}$.

**Figure 3:**
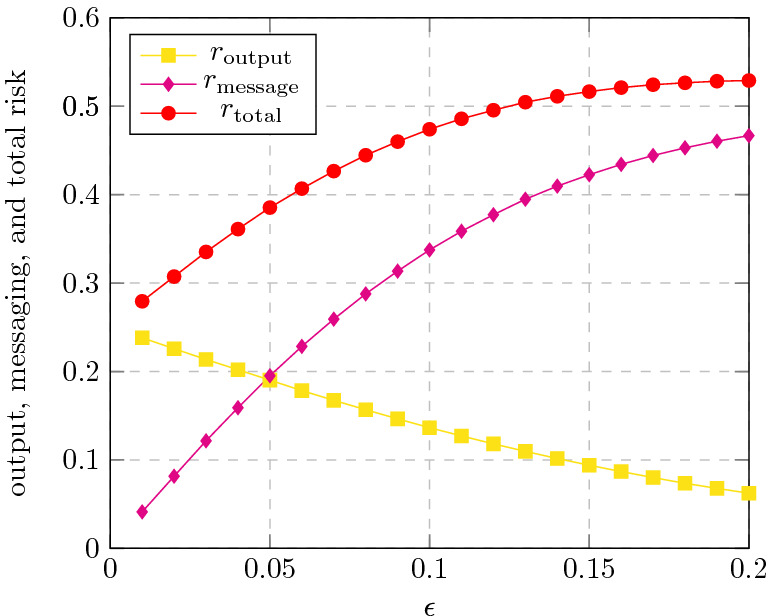
Private communication: The risk of the Quorum-Sensing protocol. The magenta plot depicts the message risk $r_\text {message}$ incurred by a rebel running the Quorum-Sensing protocol in the private communication model, when facing the Reverse police protocol. See more details in “Methods”. As in Fig. 2, the output risk $r_\text {output}$ (yellow plot) denotes the percentage of rebels which output “many” when $\rho =0.2$ (i.e, “few rebels”), and $r_\text {total}$ (red plot) is simply the sum of $r_\text {output}$ and $r_\text {message}$. The simulations should be compared with the simulations shown in Fig. 2b.

To compare with the simulations of the Quorum-Sensing protocol on the Facebook sub-network in Fig. 2a,b, we simulated this protocol on the same network, but under private communication instead of public communication. Confronting the rebels, we used the *Reverse police protocol*, which intuitively uses the rebel’s decision protocol to decide when to arrest an agent. The results of the simulations are presented in Fig. 3. As expected, the output-risk is the same as in the public communication model since the signals outgoing from an agent follow the same distribution in both models, and hence, the rebels’ output follows the same distribution. For the same reason, the percentage of rebels which output “many” in case $\rho \ge 0.8$, and the success probability are the same as under the public communication model, as presented in Fig. 2a. However, the relative message-risk and, hence, the total risk of a rebel are significantly higher under private communication than the ones observed under public communication (see Fig. 2b), despite the fact that the latter are obtained against any police protocol. Indeed, since the success probability is very close to zero when $\varepsilon \le 0.0684$ (inset in Fig. 2a), the interesting cases are when $\varepsilon >0.0684$. In this range, the total risk under private communication is at least 0.4, which is about twice the total risk under the public communication model for the same range of $\varepsilon$.

### The *Self-Immolation* protocol

As mentioned earlier, there is no deterministic uniform rebel protocol in the private communication model that is both efficient and secure. Nevertheless, we show that in this model, there exists a randomized uniform protocol, termed *Self-Immolation*, that achieves both high success probability and low risk. Importantly, however, the low risk is guaranteed only *on average*, and, in fact, the success probability of this protocol depends on few rebels that “consciously sacrifice” themselves, in the sense that they send messages that would clearly distinguish them from obedient citizens, and consequently put them at a very high risk. The proposed protocol is inspired by several historical events corresponding to the emergence of revolutions, including the self-immolation of Mohamed Bouazizi on 17 December 2010 relating to the Tunisian revolution, and the self-immolation of Thích Quang Duc on 11 June 1963, related to the Buddhist crisis in Vietnam.

The Self-Immolation protocol is as follows. Each rebel tosses a coin, and with probability *q* sends a huge number $M=\infty$ to all of its neighbors; otherwise, with probability $1-q$, it sends the message 0. In turn, at the end of the communication round, a rebel *i* outputs “many” if and only if (1) its degree is $\Delta _i\ge \Delta$ and (2) it sees more than $\tau \cdot \frac{\Delta _i}{\Delta }$ messages containing *M*, for some threshold $\tau$.

Recall that our definitions of success probability and risk were defined for deterministic protocols, where probabilities are taken with respect to the randomness in the noises and the initial selection of rebels. In this section we consider a randomized protocol, and use the notion of average risk — this is defined as the expected risk, where the expectation is with respect to the randomness of the protocol, in addition to the aforementioned sources of randomness. (Similarly, the notion of success probability also takes into account all sources of randomness, including the ones generated by the algorithm).

The next theorem states that in the private communication model, the Self-Immolation protocol achieves both high success probability and low average risk. Intuitively, the low risk follows by setting *q*, namely, the probability a rebel sends a large message, to be very low. The high success probability follows from the idea that if a rebel sees a very large number then, since this event is so rare, there should be many rebels. The formal proof of the theorem is given in the SI, Sect. F.

#### Theorem 4

Consider the Self-Immolation protocol with $q=c\log n/\Delta$, and $\tau = (c\log n)/2$ for a sufficiently large constant *c*. Consider a network such that $\Delta \gg \log n$. Then, the average risk is $O(\log n/\Delta )$ and the success probability is $1-O(1/n^2)$.

## Discussion and future works

This paper argues that the communication infrastructure can play a significant role in rebels’ ability to estimate their fraction in the population securely. Our main takeaway message is that even under extreme surveillance conditions, there are simple deterministic protocols in the public communication model that allow rebels to estimate their fraction in the population while keeping a negligible risk of each rebel being identified as such. Importantly, these results hold assuming that the median degree in the network is sufficiently large, which happens for some social media networks but not for all. For example, the median degree of the Facebook network is significantly higher than the logarithm of the number of participants, which allowed our simulations to work effectively. In contrast, the median in-degree of Twitter is close to 1^34^, implying that our quorum-sensing protocols will not function well in this network. This suggests that in the context of secretly estimating the support for a revolution, Facebook might be more effective than Twitter.

Public communication can also capture communication in crowd assemblies such as the one that gathered during Ceauescu’s last speech (as discussed in “Introduction”). Among other possible explanations, our results suggest that the emergent changeover in the crowd’s behavior, from being completely submissive to rebellious, can be partially attributed to the high density of the gathered crowd, which in some sense increases the degree of individuals, when viewed as participants in a public communication network. In this sense, it may be reasonable to assume that if the assembly was significantly less crowded and more spatially sparse, perhaps such a changeover would not have occurred.

The principle revealed in this paper is in fact not limited to overthrows of dictatorships by rebels and can be pertinent to other social movements in which the participating individuals prefer to remain covert. Interestingly, yet more speculatively, our setting may further find relevance in the microbiological world. Indeed, quorum-sensing mechanisms are known to be utilized by bacteria communities to identify when their density passes a certain threshold^12,26,35–37^, e.g., before attacking a host tissue^12^. Moreover, communication between bacteria follows a diffusion process of autoinducers, which is, in some sense, reminiscent of public communication. In the presence of the immune system, it is plausible that pathogenic bacteria act covertly, especially while being surrounded by non-pathogenic bacteria communities. In this context, our results may suggest that in order to perform the quorum-sensing covertly, such bacteria would avoid using distinct autoinducers in their signaling, and instead, use signals composed of a mixture of molecules types that are already used by nearby nonpathogenic bacteria, while slightly biasing their proportions. A supporting empirical evidence is the fact that several common species of bacteria, including *B. subtilis*, *V. harveyi*, and its pathogenic relative, *V. cholerae*, have been shown to utilize different combinatorial combinations of autoinducers which are used (either separately or in other combinations) by other bacteria^35,36,38–42^. Explanations for the use of multiple autoinducers have been given using arguments from evolutionary game theory^40,43^. The current paper suggests that this phenomena, and particularly the overlap in the autoinducer combinations, could also be explained in the context of covert communication. Further research is required to inspect this direction of research concerning bacterial quorum-sensing. In particular, to the best of our knowledge, it is currently unknown whether the immune system is actually able to detect the source of the autoinducers secreted during quorum-sensing.

The current paper introduces the concept of covert multi-party computation under natural computational conditions. In this preliminary work we have focused on the basic aspects of the model and it is of interest to further inspect other natural aspects. Below we list several possibilities for extending our model, suggested as future work.

First, real-life networks are inherently heterogeneous in various aspects. In the SI, Sect. A, we study one manifestation of heterogeneity, in which nodes have different “strengths”. Intuitively, a “stronger” node has a higher social impact, and hence, a revolution may have higher chances to succeed if many strong rebels join it. Another aspect of heterogeneity concerns having connections with varying strengths. In our context, this may represent reliability factors (i.e., a message arriving from a strongly-connected neighbor may appear as more reliable than a message arriving from a weakly-connected neighbor). We believe that, in some cases, the node-weighted approach can be adapted to handle the edge-weighted approach, however, this direction of research requires further investigation.

Another interesting aspect that is neglected in the current work concerns a priori knowledge of agents regarding the rebel distribution. This can include partial knowledge regarding $\rho$, i.e., the fraction of rebels in the population, or the case that the behavior type of an agent is known to certain other agents, and not merely to itself. It is important to note that, in general, such a priory knowledge can only provide an advantage to the rebels, since estimating the fraction of rebels is expected to be easier when having partial knowledge regarding their distribution compared to the case of having no such knowledge (as studied here). Hence, considering such a priori knowledge can only improve the (already highly efficient) protocols we consider for public communication. Since the main goal of this paper is to demonstrate efficiency under public communication, we do not consider such a priori knowledge assumptions. On the other hand, we note that adding such assumptions is expected to complicate the lower bound proof regarding private communication (Theorem 3). Hence, studying the impact of such a priori knowledge assumptions in the private communication model may lead to interesting theoretical results. This direction of research is left for future work.

Finally, it remains open whether a lower bound result for private communication, in the spirit of Theorem 3, holds also when the communication is allowed to consist of several rounds. Indeed, in this work we restricted attention to a single round of parallel communication, which was sufficient for our algorithms operating under the public communication model to execute both correctly and securely. Contrasting these upper bounds, Theorem 3 provides a lower bound result under the private communication model, which holds when the communication is restricted to a single parallel round. The question of whether or not a similar lower bound result can be proved under multiple rounds it left for future work.

## Methods

### Asymptotic notation

We use the classical Bachmann–Landau notation to denote the limiting behaviour of functions as their arguments, which are in our case the size of the population *n* and the median degree $\Delta$, tend to infinity. Specifically, consider two non-negative functions *f* and *g* defined on $N\times N$. The “*O*” notation stands for an upper bound in following sense. We say that $f(n,\Delta ) \in O(g(n,\Delta ))$ if there exists a positive real $c > 0$ and two integers $n_0$ and $\Delta _0$ such that $f(n,\Delta ) \le c \cdot g(n,\Delta )$ for every $n \ge n_0$ and every $\Delta \ge \Delta _0$. Conversely, the notation “$\Omega$” represents a lower bound in the following sense. We say that $f(n,\Delta ) \in O(g(n,\Delta ))$ if there exists a positive real $c > 0$ and two integers $n_0$ and $\Delta _0$ such that $c \cdot g(n,\Delta ) \le f(n,\Delta )$ for every $n \ge n_0$ and every $\Delta \ge \Delta _0$.

Finally, the notation “*o*” allows us to state that a function *g* grows much faster than another function *f*, as their arguments increase. In Theorem 2 we consider the argument of the function being the median degree $\Delta$. Specifically, we say that $f(\Delta ) \in o(g(\Delta ))$ if, for every positive real $c > 0$, there exist an integer $\Delta _0$ such that $f(\Delta ) \le c \cdot g(\Delta )$ for every $\Delta \ge \Delta _0$. In particular, if $g(\Delta )$ is a constant function, then we have that $f(\Delta )$ is asymptotically smaller than any constant value (that is, for every positive real $c > 0$, there exist an integer $\Delta _0$ such that $f(\Delta ) \le c$ for every $\Delta \ge \Delta _0$). In this case, we write $f(\Delta ) \in o(1)$.

### Mathematical proofs

Most of our analysis consists of mathematical proofs, proving upper and lower bounds on the performances of algorithms in rigorous manner. The proofs extensively use the following well-known bounds from probability theory.

*Union bound* For any events $E_1,\ldots ,E_n$, $P\left( \bigcup _{i=1}^nE_i\right) \le \sum _{i=1}^n P(E_i)$.

*Markov inequality* If *X* is a non-negative random variable, then, for any real value $\delta > 0$,

$$\begin{aligned} P(X \ge \delta ) \le \frac{E[X]}{\delta }. \end{aligned}$$

*Chernoff bound for a normal distribution* If *X* is a random variable drawn from $N(\mu ,\sigma ^2)$, then, for all $x>0$,

1 $$\begin{aligned} P(\mid X-\mu \mid \ge \sigma x)\le 2 e^{-x^2/2}. \end{aligned}$$

*Chernoff bounds for sum of Boolean random variables* Suppose $X_1,\ldots , X_n$ are independent random variables taking values in $\{0, 1\}$. Let *X* denote their sum and let $\mu = E[X]$ denote the sum’s expected value. Then for any $0\le \delta \le 1$,

2 $$\begin{aligned} P(X\le (1-\delta )\mu )\le e^{-\frac{\delta ^2\mu }{2}}, \end{aligned}$$

and

3 $$\begin{aligned} P(X\ge (1+\delta )\mu )\le e^{-\frac{\delta ^2\mu }{3}}. \end{aligned}$$

**Figure 4:**
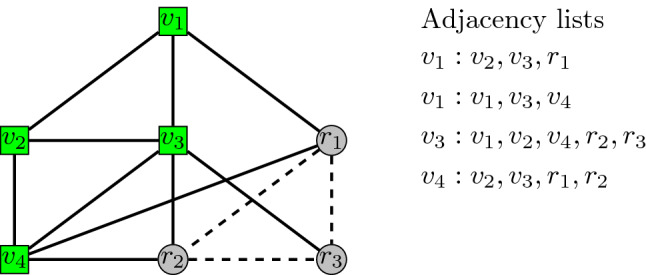
Visited and reached nodes in the sample of the Facebook social graph. We give an example of how our simulations treat differently the visited and reached nodes, as defined by the Breadth-First-Search (BFS) crawling on the Facebook social graph^28,29^. In the example, the visited nodes, that is, the nodes completely explored by the BFS are $v_{1}$, $v_{2}$, $v_{3}$, and $v_{4}$ (green squares), while the reached nodes, that is, the nodes only reached by the BFS are $r_{1}$, $r_{2}$, and $r_{3}$ (gray circles). All edges are unweighted and denote the fact that the two endpoints of an edge are friends in the Facebook social graph. However, while the edges incident to visited nodes (solid lines) are included in the network used in our simulations, the edges between two reached nodes (dashed lines) are not considered (even if the corresponding two Facebook users are friends). On the right, we show the adjacency lists that would be used in our simulations with regards to the aforementioned example: As it can be seen, there is no adjacency list for the reached nodes, since the neighborhood of these nodes has not been fully explored.

### Simulations of the Quorum-Sensing protocol

**Figure 5:**
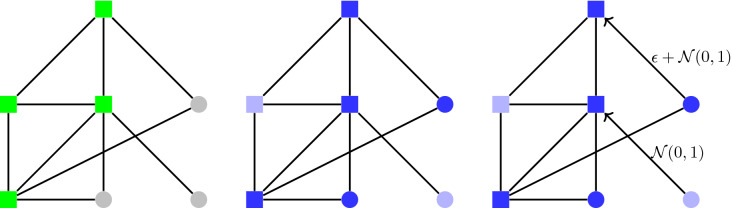
Reached nodes and sending phase of the Quorum-Sensing protocol. How the reached nodes participate to the sending phase of the Quorum-Sensing protocol (reached nodes are the gray circles on the left). Similarly to visited nodes (green squares), reached nodes can be either obedient citizens or rebels: for example, on the center, we have two reached nodes which are rebels (dark blue circles) and one which is an obedient citizen (light blue circle). As any other node of the network, a rebel reached node plays the sending phase of the Quorum-Sensing protocol and sends to each of its neighbors (which is a visited node) a value distributed by $\varepsilon +{\mathcal {N}}(0,1)$, while an obedient citizen reached node sends to each of its neighbors (which is a visited node) a value distributed by ${\mathcal {N}}(0,1)$ (see on the right). Only the rebel visited node will execute the detecting phase of the protocol in order to decide whether to output “many”. Hence, the success probability has to refer to fraction of rebel visited nodes that output “many”, among total rebel visited nodes.

We simulated the Quorum-Sensing protocol on the sample of the Facebook social graph released in^28,29^, which contains approximately 1.2 million Facebook users reached by one breadth-first-search traversal. Actually, in^29^ several candidate graph-crawling techniques are compared in terms of their sampling bias and efficiency: the breadth-first search is one of these techniques, and it has been repeatedly used for sampling online social networks, even though it is known that it leads to samples that are somewhat biased^29^. The reason why we used the sample based on breadth-first search is simply the fact that this sample is, as far as we know, one of the largest and latest samples of the Facebook social graph publicly available on the web (crawling the Facebook graph in order to obtain a more recent sample is beyond the scope of this paper). More precisely, the network contains 61,876,615 nodes (that is, Facebook users) which have been reached by the breadth-first-search. Among these, 1,189,767 nodes (called *visited nodes*) have also been explored, so that their entire friendship neighborhood is included in the network. For the remaining nodes (called *reached nodes*), the network includes only their connections to the visited nodes (see Fig. 4 for a visualization, where the green squares represent visited nodes, and the gray circles represent reached nodes). For this reason, the median degree of the network will be computed only by referring to the visited nodes, that is, the nodes whose degree has been exactly computed. In the case of the sample of the Facebook social graph, the median degree (of the visited nodes) is 185, which is quite high when compared to $\log _2(1{,}189{,}767)\approx 20$. For the very same reason, a reached node (which can be either an obedient citizen or a rebel) will play only the sending phase of the protocol (see Fig. 5). Of course, this implies that, while computing the success probability, we have to refer only to the fraction of rebels among the visited nodes which output “many”.

Concerning the detecting phase of the protocol, recall that it will be played by a rebel visited node only if its degree is not lower than the median degree (in our case, 185). If this is case, the rebel visited node will compute the average of the received signals (including the ones arrived by its neighbors which are reached nodes) and it will output “many” if and only if this average is at least $\varepsilon /2$. The results shown in Figs. 2a,b, and 3 were obtained by running 10 experiments, apart from the inset in Figure 2a, which has been obtained by running 100 experiments.

Finally, in order to compute the message risk $r_{message}$ in Fig. 3, in the case of the private communication model, we simulated the Reverse police protocol with respect to visited nodes only (rebel or obedient citizen). That is, for each visited node *u* whose degree is at least as large as the median degree, the police protocol computes the average of the signals sent by *u* to all its neighbors (including the ones sent to its neighboring reached nodes) and arrests *u* if and only if this average exceeds $\varepsilon /2$.

### Simulations with homophily

According to^31^, the dyadicity *D* of a network is defined as the ratio between the number of rebel/rebel edges which are in the network and the expected number of such edges if the edges of the networks were created randomly in an independent manner. For example, in the case shown in the center of Fig. 5, we have $n=7$ nodes and $m=10$ edges, hence, the probability that two nodes are connected is $p=\frac{m}{n(n-1)/2}=\frac{10}{21}$. Since the number of rebel nodes is $n_{\text {rebel}}=5$, the expected number of rebel/rebel edges is equal to $\mu _{\text {rebel/rebel}}=\frac{n_{\text {rebel}}(n_{\text {rebel}}-1)}{2}p=\frac{100}{21}$. On the contrary, in the figure we have that the number of rebel/rebel edges is equal to $m_{\text {rebel/rebel}}=6$. Hence, the dyadicity of the network is equal to $D=\frac{m_\text {rebel/rebel}}{\mu _{\text {rebel/rebel}}}=\frac{6}{100/21}=1.26$.

In order to produce Fig. 2c,d, we conducted simulations over a sample of the Facebook social graph using different levels of dyadicity. Note that in this case, as previously mentioned, we have to consider that there are no edges connecting the reached nodes (that is, the edges are not equally distributed). For this reason, we can modify the original definition of dyadicity given in^31^ as follows. Let $n_{v}$ (respectively, $n_{r}$) be the number of visited (respectively, reached) nodes, $v_{\text {rebel}}$ (respectively, $r_{\text {rebel}}$) be the number of rebel visited (respectively, reached) nodes, and $m_{vv}$ (respectively, $m_{vr}$) be the number of visited/visited (respectively, visited/reached) edges. The probability that two visited nodes are connected is equal to $p_{vv}=\frac{m_{vv}}{n_v(n_v-1)/2}$, while the probability that a visited node and a reached node are connected is equal to $p_{vr}=\frac{m_{vr}}{n_vn_r}$. The expected number of rebel/rebel edges is defined as $\mu _{\text {rebel/rebel}}=\frac{v_{\text {rebel}}(v_{\text {rebel}}-1)}{2}p_{vv}+v_{\text {rebel}}r_{\text {rebel}}p_{vr}$, and the dyadicity of the network is then defined as $D=m_{\text {rebel/rebel}}/\mu _{\text {rebel/rebel}}$, where $m_{\text {rebel/rebel}}$ is the number of rebel/rebel edges in the network. Again referring to the labeled network shown in the center of Fig. 5, we have that $n_v=4$, $n_r=3$, $v_{\text {rebel}}=3$, $r_{\text {rebel}}=2$, $m_{vv}=5$, and $m_{vr}=5$. Hence, $p_{vv}=\frac{5}{6}$, $p_{vr}=\frac{5}{12}$, and $\mu _{\text {rebel/rebel}}=5$. Since $m_{\text {rebel/rebel}}=6$, we have that the dyadicity of the network is equal to $D=m_{\text {rebel/rebel}}/\mu _{\text {rebel/rebel}}=1.2$ (slightly smaller than the previously computed dyadicity).

#### Generating homophily

The problem, now, is how to assign rebel/citizen labels to the nodes of the sample of the Facebook social graph in order to obtain different levels of dyadicity, that is, different values of *D*. This problem is, in general, NP-complete^44^. In our simulations, we designed a *labeling algorithm* to obtain dyadicity of roughly *D*, while maintaining that the fraction of rebels is approximately $\rho$. Our algorithm depends on $\rho$ and three parameters *q*, *p*, and *h* (standing for homophily), such that $0\le q,p,h\le 1$. Specifically, with probability *q* the algorithm chooses an unlabeled visited node uniformly at random (from all unlabeled visited nodes), and then, with probability *p*, it assigns it the rebel label (this branch of the algorithm does not produce homophily). Otherwise (that is, with probability $1-q$), it chooses uniformly at random a rebel node (visited or reached) and then “propagates” the rebel label to one of its neighbors with probability *h*. Formally, the labeling algorithm is the following one.

- Repeat until all visited nodes are labeled and there are no rebel labeled nodes with unlabeled neighbors:
  - With probability *q* do: Choose uniformly at random an unlabeled visited node *u* and, with probability *p*, assign the rebel label to *u* (otherwise assign to *u* the obedient citizen label).
  - Otherwise (that is, with probability $1-q$) do: Choose uniformly at random a rebel labeled node *u* (among the rebel labeled nodes) and an unlabeled neighbor *v* of *u*. Then, with probability *h*, assign the rebel label to *v* (otherwise assign to *v* the obedient citizen label).
- Repeat until a fraction $\rho$ of all nodes are rebel labeled:
  - Choose an unlabeled (reached) node uniformly at random, and assign to it the rebel label.

Note that, due to the fact that the number of visited nodes is small with respect to the number of reached nodes, the second loop is necessary since, at the end of the first loop, we are not guaranteed that a fraction $\rho$ of all nodes has been labeled as rebels.

#### Experimenting with homophily

The entire sample of the Facebook social graph network is too large to apply the previously described labeling algorithm in a reasonable amount of time. For this reason, we have worked with a sample of this sample, obtained by considering only the first 100,000 visited nodes, which induce 13,717,003 reached nodes (the median visited node degree in this new network is 176). By applying the labeling algorithm with parameters $\rho =0.8$, $q=0.25$, $p=0.5$, and $h=0.9$, we obtained a network with dyadicity $D\approx 1.2$ and having approximately $80\%$ of nodes being rebels, and by applying the labeling algorithm with parameters $\rho =0.2$, $q=0.45$, $p=0.1$, and $h=0.2$, we obtained a network with dyadicity $D\approx 1.2$ and approximately $20\%$ of the nodes being rebels. In Fig. 2c we show the behavior of the Quorum-Sensing protocol applied to these two labeled networks. In Fig. 2d, instead, we show the behavior of the Quorum-Sensing protocol applied to two labeled networks with dyadicity $D\approx 1.7$ (obtained by using the labeling algorithm with parameters $\rho =0.6$, $q=0.1$, $p=0.2$, and $h=1.0$, and $\rho =0.4$, $q=0.3$, $p=0.4$, and $h=0.7$).

At this point, we would like to mention a small technical issue regarding the simulation associated with Fig. 2d. In general, it may become impossible to construct a network that has both many rebels and a large dyadicity value. In particular, when $\rho =0.8$ one cannot achieve dyadicity as large as $D=1.7$. Hence, in order to obtain this high dyadicity in presence of many rebels, we reduced the value of $\rho$ in the case of “many rebels” from 0.8 to 0.6, and, for symmetry considerations, we also increased the value of $\rho$ in the case of “few rebels” from 0.2 to 0.4.

## Supplementary Information

Supplementary Information 1.
