# Supplementary material for: Public communication can facilitate low-risk coordination under surveillance
Source: Sci Rep. 2022 Mar 2;12:3433. doi: 10.1038/s41598-022-07165-9 (PMC8891294; doi:10.1038/s41598-022-07165-9)
Supplement: Supplementary file 1 — Supplementary Information 1. [file 41598_2022_7165_MOESM1_ESM.pdf]

Supplementary Information

A Simulations with synthetic networks

As mentioned in the description of the simulations in Section 4, the sample of the Facebook social graph has been obtained by performing a breadth-first search on the Facebook network in the year 2009. As far as we know, this sample is the largest latest sample of the Facebook social graph which is publicly available on the web. More recent samples of the Facebook social graph have been studied in[1], however, the authors explicitly say in the associated web site that they "cannot distribute the graphs for obvious reasons". Nevertheless, the degree distribution of few of these samples are publicly available. Hence, we have performed our experiments also on "synthetic" networks obtained by applying the Chung-Lu model[2] over the degree distribution of one specific sample of the Facebook social graph (Figure 6(a)).

We recall that, given a degree distribution $d_1, \ldots, d_n$ of n nodes, a graph generated according to the Chung-Lu model includes an edge between two nodes u and v with probability $\frac{d_u d_v}{D}$, where $D = \sum_{i=1}^{n} d_i$. We have generated a random graph by using the degree distribution of the Swedish Facebook sample in 2011, which is available on the web site of the Laboratory for Web Algorithmics of the University of Milan (see `https://law.di.unimi.it`). The generated graph has 4,345,050 nodes and its median degree is 101 (actually, we have generated several random graphs by using the same degree distribution, and the experimental results are consistently the same). In order to efficiently generate a random graph with this large number of nodes, we used the grass-hopping technique described in[3]. The results of the application of the Quorum-Sensing protocol to this random graph are shown in Figure 6(a). As it can be seen, they are quite similar to the results obtained in the case of the sample of the 2009 Facebook social graph obtained by performing a breadth-first search (Figure 2). Indeed, the minimum value of the total risk r_{total} is approximately 0.22, compared to approximately 0.2, corresponding to the case of the sample of the Facebook social graph. Moreover, this value is obtained at roughly $\varepsilon = 0.14$, compared to roughly $\varepsilon = 0.16$, corresponding to the case of the sample of the Facebook social graph. In both cases, this corresponds to a success probability of 1.

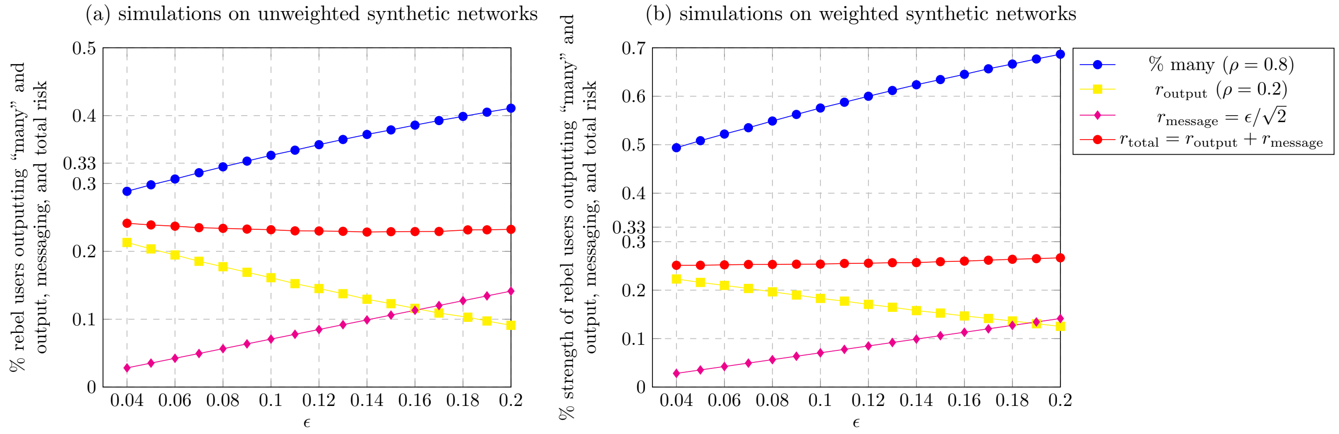

Figure 6. Public communication: Simulations on unweighted and weighted synthetic networks. We ran 10 times the Quorum-Sensing protocol (a) and the weighted Quorum-Sensing protocol (b) on the network generated by using the Chung-Lu model with the degree distribution of the 2011 Swedish Facebook graph. In both (a) and (b), the blue circle plot depicts the success probability when $\rho = 0.8$. Specifically, on (a), it depicts the (average) percentage of rebels which output "many" when $\rho = 0.8$, while on (b), the blue circle plot depicts the success probability in the weighted case (see Eq. (4)), when $\rho = 0.8$. On both figures, the yellow square plot depicts the output risk r_{output}, that is, the fraction of rebels that output "many" given that $\rho = 0.2$. The magenta diamond plot depicts the message risk r_{message} which equals to $\varepsilon/\sqrt{2}$ (since a rebel sends the message ε, see Lemma 1). Finally, the red circle plot depicts the total risk r_{total} which is defined as the sum of r_{output} and r_{message}.

Simulations on networks with nodes of different "strengths". In addition, we used the random graph generated according to the Chung-Lu model to perform simulations on heterogeneous networks, whose nodes have different "strengths" (Figure 6(b)). Intuitively, a "stronger" node has more impact when initiating a revolution, and hence, a revolution has more chances to succeed if many strong rebels join it. Clearly, there are numerous ways of associating strengths to nodes, and it is hard to evaluate which one captures real-life scenarios. Here, we decided to associate to each node j a weight w_j that equals to its degree. (Note that such a weight assignment cannot be done in the case of the sample of the 2009 Facebook social graph, since the degree of the reached nodes is not known). Since we assume that "stronger" rebels contribute more to the success of the revolution we

modify the definition of success probability requiring that at least one third of the "total strength of the rebels" is involved in the revolutionary movement. Formally, the *weighted success probability* is defined as the probability that

$$\frac{\sum_{u\in V_{\text{many}}} w(u)}{\sum_{u\in V_{\text{rebel}}} w(u)} \geq \frac{1}{3}, \tag{4}$$

where V_{rebel} (respectively, V_{many}) denotes the set of rebels (respectively, the set of rebels that output "many"). In contrast to the success probability, the risk factors are not affected by the weights. In particular, the output risk still refers to the fraction of rebels which output "many", regardless of their weight.

In order to deal with the case of weighted nodes, the detecting phase of the Quorum-Sensing protocol has been modified in the following way. A rebel j outputs "many" if and only if

1. the degree of j is at least the median degree in the network (even in presence of weights, we still want that the decision of a rebel is taken on the ground of many signals), and
2. the weighted arithmetic mean of the signals received by j from its neighbors is at least $\varepsilon/2$, that is, $\frac{\sum_{i\in N(j)} w_i s_{i,j}}{\sum_{i\in N(j)} w_i} \geq \frac{\varepsilon}{2}$.

The results of the application of this weighted variation of the Quorum-Sensing protocol to the random graph generated with the Chung-Lu model are shown in Figure 6(b). As it can be seen, in this case the minimum total risk r_{total} is a slightly increasing function of ε: its minimum value is approximately 0.22 and it is obtained at $\varepsilon = 0.04$, which corresponds to a (weighted) success probability of 1.

B Two simple applications of Chernoff's inequality

As applications of Equations 2 and (3), we prove two claims, which will be used later.

Claim 1. *Let G be a graph with median degree Δ. If $\rho \geq 0.8$, then, for n sufficiently large, with high probability (namely, at least $1 - 1/n^2$), the number of rebels with degree at least Δ is at least $n/3$.*

Proof. We consider a graph G with median degree Δ. For the sake of simplicity, we assume that the number of agents whose degree is at least Δ is exactly $n/2$. For any agent u whose degree is at least Δ, let X_u be the random binary variable indicating whether u is a rebel. We have $P(X_u = 1) = \rho$. Let $X = \sum X_u$. Then

$$\mu = E[X] = \sum E[X_u] = \rho n/2.$$

By applying Equation 2 with $\delta = 1 - 2/(3\rho)$, we have that

$$P(X < n/3) \leq e^{-\frac{\left(1-\frac{2}{3\rho}\right)^2 n\rho}{4}}.$$

In order to have that $P(X < n/3) < \frac{1}{n^2}$, we need that

$$e^{\frac{\left(1-\frac{2}{3\rho}\right)^2 n\rho}{4}} > n^2 \Leftrightarrow \frac{\left(1-\frac{2}{3\rho}\right)^2 n\rho}{4} > \log n^2 \Leftrightarrow \left(1-\frac{2}{3\rho}\right)^2 \rho > \frac{4\log n^2}{n}.$$

If $\rho \geq 0.8$, the latter inequality is satisfied, for n sufficiently large. □

Claim 2. *Consider the case $\rho = 0.2$. For any $\delta > 0$ there exists a constant c, such that if $\Delta_u \geq \Delta > c\log n$, then the probability that the number of rebels among the neighbors of u is within the interval $[(1-\delta)\rho\Delta_u, (1+\delta)\rho\Delta_u]$ is at least $1 - \frac{1}{n^2}$.*

Proof. Let X_i be the random variable indicating whether neighbor i is a rebel ($X_i = 1$) or not ($X_i = 0$). Since every agent is chosen to be a rebel with probability ρ independently of the others, the X_i are mutually independent variables. Let X be the number of rebels among the neighbors of u. Then $X = \sum_{i=1}^{\Delta_u} X_i$. The expected value of X is $\rho\Delta_u$. Therefore, by Eq. (2), for any fixed $0 \leq \delta \leq 1$ and if $\Delta_u \geq \Delta > c\log n$ (recall that $\rho = 0.2$), we have

$$P(X < (1-\delta)\rho\Delta_u) < e^{-\frac{\delta^2\rho\Delta_u}{2}} = e^{-\frac{\delta^2\Delta_u}{10}} < e^{-\frac{\delta^2 c}{10}\log n},$$

and by Eq. (3),

$$P(X < (1+\delta)\rho\Delta_u) < e^{-\frac{\delta^2\rho\Delta_u}{3}} = e^{-\frac{\delta^2\Delta_u}{15}} < e^{-\frac{\delta^2 c}{15}\log n},$$

Therefore, we can take c to be a sufficiently large constant (for example, $c > \sqrt{\frac{60}{\delta}}$), so that (if $n > 1$) both $e^{-\frac{\delta^2 c}{10}\log n}$ and $e^{-\frac{\delta^2 c}{15}\log n}$ would be smaller than $1/2n^2$, concluding the proof of the claim. □

C Analysis of the Quorum-Sensing protocol

The goal of this section is to prove Theorem 1. Recall that the theorem considers the public communication model and a network with median degree Δ, and a fixed $\varepsilon > 0$. The goal is to show that for any sufficiently large n and $\Delta > c \log n$ for a sufficiently large constant c, the success probability of the Quorum-Sensing protocol is at least $1 - O(1/n^2)$, while the total risk is at most 0.715ε.

Towards this, we first obtain the following lemma that establishes the low relative message-risk of the Quorum-Sensing protocol. Intuitively, its proof is based on first showing that the "distance" between the distributions of the signals whose mean is ε (corresponding to the messages out-going from a rebel) versus those whose mean is 0 (corresponding to the messages out-going from an obedient citizen) is small, and then deducing that any police protocol that attempts to distinguish them must make many mistakes. In order to prove this we rely on concepts and techniques adopted from the area of *statistical hypothesis testing*.

Lemma 1. *Consider the public communication model, and assume that each rebel announces the message $\varepsilon > 0$. Any police protocol that arrests a rebel with probability at least δ, must arrest an obedient citizen with probability at least $\delta - \varepsilon/\sqrt{2}$. In other words, the relative message-risk of a rebel is at most $\varepsilon/\sqrt{2}$. This holds with respect to any underlying graph.*

Proof. Recall that each obedient citizen sends the number 0 which is then received by the police as a signal $a \sim N(0,1)$, distributed normally with mean 0 and variance 1. A rebel sends the message ε which will in turn be received by the police as a signal $b \sim N(\varepsilon, 1)$. Our goal is to prove that any police protocol that arrests a rebel with probability at least δ, must arrest an obedient citizen with probability at least $\delta - \varepsilon/\sqrt{2}$. Intuitively, to this aim, we will show that the "distance" between the signal distribution of an obedient citizen and that of a rebel is small, and then deduce that any police protocol that attempts to distinguish them must make many mistakes.

To formalise the notion of distance between two distributions, we use two measures which are standard in the field of statistical hypothesis testing. Given two distributions P_0 and P_1 supported on the real numbers, we define the following.

- *Total variation distance*

$$\mathrm{TV}(P_0, P_1) := \frac{1}{2} \int_{-\infty}^{\infty} |p_0(x) - p_1(x)| dx$$

- *Kullback-Leibler divergence*

$$\mathrm{KL}(P_0, P_1) := \int_{-\infty}^{\infty} p_0(x) \log \frac{p_0(x)}{p_1(x)} dx$$

Here, $p_i(x)$ stands as a shorthand for $P_i(X = x)$, for $i = 0, 1$, and the logarithm is the natural one.

A configuration of messages C is a vector of messages, $C = (m_1, m_2, \ldots, m_n)$, where m_i is the message sent by agent i. Consider any police protocol g. Being liberal, we assume that the police can decide whether to arrest an individual i, based on all signals it receives from all agents, and not only on the one corresponding to the outgoing message from i. Hence, g can be viewed as a (possibly probabilistic) function $g : R^n \to \{0,1\}^n$, deciding for each possible set of signals whether to arrest agent i, with $1 \le i \le n$. Fix an agent i and fix the messages $C_{\neq i} = (m_1, m_2, \ldots, m_{i-1}, m_{i+1}, \ldots, m_n)$ sent by all other agents. Let $g_{i, C_{\neq i}} : R \to \{0,1\}$ be the restriction of g to agent i, given that all others sent $C_{\neq i}$. For brevity, we omit the subscripts i and $C_{\neq i}$. Let X be a random variable representing the value of the signal received by the police corresponding to the message sent by agent i, i.e., X is drawn from either P_0 or P_1. If the police believes that X corresponds to a message sent by an obedient citizen, then $g(X) = 0$ (and the sender i is not arrested), and if it believes that X corresponds to a message sent by a rebel, then $g(X) = 1$ (and i is arrested). Our goal is to show that g necessarily makes many mistakes.

The next lemma shows that if the total variation distance between P_0 and P_1 is small, then any police function g can distinguish obedient citizens from rebels only with a very small probability.

Lemma 2 (Neyman-Pearson [4, Lemma 4.3 and Proposition 4.4])**.** *Let P_0 and P_1 be two distributions with support R. Let X be a random variable drawn from either P_0 or P_1. Consider a (possibly probabilistic) mapping $g : R \to \{0,1\}$ that attempts to "guess" whether the observation X was drawn from P_0 (in which case it outputs 0) or from P_1 (in which case it outputs 1). Then, we have that*

$$P_0(g(X) = 1) + P_1(g(X) = 0) \ge 1 - \mathrm{TV}(P_0, P_1),$$

where $P_i(g(X) = j)$ stands for $P(g(X) = j \mid X \in P_i)$, for $i, j \in \{0,1\}$.

Lemma 2 bounds the probability of both kinds of "wrong" police actions: the arrest of an obedient citizen, and the non-arrest of a rebel. Our goal now is to bound from above the total variation distance of the two distributions considered. To this end, we use the next connection between the total variation distance and the Kullback-Leibler divergence.

Lemma 3 (Pinsker [4, Lemma 4.8])**.** *For any two distributions P_0 and P_1,*

$$\mathrm{TV}(P_0,P_1) \le \sqrt{\mathrm{KL}\,(P_0,P_1)}.$$

As, in our case, P_0 and P_1 are normal distributions, computing their Kullback-Leibler divergence is easy:

$$\mathrm{KL}(P_0,P_1) = \int_{-\infty}^{\infty} \frac{1}{\sqrt{2\pi}} \exp(-x^2/2) \log \frac{\exp(-(x-\varepsilon)^2/2)}{\exp(-x^2/2)} dx = \varepsilon^2/2.$$

By Lemma 3, it follows that $\mathrm{TV}(P_0,P_1) \le \varepsilon/\sqrt{2}$.

Fix an execution of the protocol, and the messages of all players. For a signal received by the police from an agent, Lemma 2 implies that the probability of mistake by the police is at least $1-\varepsilon/\sqrt{2}$, i.e., $P_0\,(g(X)=1)+P_1\,(g(X)=0) \ge 1-\varepsilon/\sqrt{2}$. If the police arrests a rebel with probability at least δ, i.e., $P_1\,(g(X)=1) \ge \delta$, then $P_1\,(g(X)=0) \le 1-\delta$. This implies that $P_0\,(g(X)=1) \ge \delta - \varepsilon/\sqrt{2}$, i.e., the police arrests an obedient citizen with probability at least $\delta-\varepsilon/\sqrt{2}$. This completes the proof of Lemma 1. □

We now continue with the proof of Theorem 1. As stated in Lemma 1, for a very small $\varepsilon > 0$, the rebel messaging protocol incurs very small relative message-risk. To complete the proof of Theorem 1 we show that the total-risk is also very small, and that, nevertheless, the effectiveness of the Quorum-Sensing protocol is extremely high. The proof is based on properly applying Chernoff's inequalities.

Consider a rebel u with degree Δ_u. To upper bound its total-risk, we need to upper bound the output-risk of u, that is, r_{output}. Recall that r_{output} is defined as the probability that u outputs "many", given that $\rho \le 0.2$. Without loss of generality, we may assume that $\rho = 0.2$, since for any $\rho < 0.2$, the probability to that u outputs "many" can only decrease.

If $\Delta_u < \Delta$, then u does not output anything and, hence, $r_{\text{output}} = 0$. Consider now the case $\Delta_u \ge \Delta$. Denote by

$$\bar{s} = \sum s_i/\Delta_u$$

the average value of the signals u receives.

Note that s_i, the signal received from neighbor i, is actually distributed according to a mixture of normal distributions and does not follow a normal distribution. However, once we know if neighbor i is a rebel or not, then s_i becomes a normally distributed (according to $N(0,1)$ if i is an obedient citizen or $N(\varepsilon,1)$ if it is a rebel). This presents a small complication in the analysis which we solve by first conditioning on the likely event that the number of rebels among the neighbors of u, is close to the expected value $\rho\Delta_u$.

Indeed, recall Claim 2, and let us condition on the highly likely event that the number of rebels among the neighbors of u is within the interval $[(1-\delta)\rho\Delta_u,(1+\delta)\rho\Delta_u]$. Consider any fixed population of rebel neighbors of u satisfying this. For this fixed population, denote by $\hat{\rho}_u$ the fraction of rebels among the neighbors of u. (Therefore, $\hat{\rho}_u \in [(1-\delta)\rho,(1+\delta)\rho]$.) Recall that we assume that $\rho = 0.2$ and let us take $\delta = 0.1$. Then $\hat{\rho}_u \in [0.18, 0.22]$.

Moreover, since we condition on a specific assignment of rebels, each variable s_i is normally distributed. This implies that $\bar{s}$ is also normally distributed according to $N(\mu,\sigma^2)$, where $\mu = \hat{\rho}_u\varepsilon \le 0.22\varepsilon$ and $\sigma^2 = 1/\Delta_u$. By applying the Chernoff bound for a normal distribution (Eq. (1)), we have that the output-risk satisfies

$$r_{\text{output}} = P\,(\bar{s} \ge \varepsilon/2) \le P(\bar{s}-\mu \ge 0.28\varepsilon) \le P\left(|\,\bar{s}-\mu\,| \ge \sigma \cdot 0.28\varepsilon\sqrt{\Delta_u}\right) < 2e^{-\frac{(0.28)^2\Delta_u\varepsilon^2}{2}} < 2e^{-\frac{\Delta\varepsilon^2}{32}}.$$

By Lemma 1, the relative message-risk $r_{message}$ is at most $\varepsilon/\sqrt{2}$. Hence, the total risk is

$$r_{\text{total}} = r_{\text{output}} + r_{\text{message}} < \frac{\varepsilon}{\sqrt{2}} + 2e^{-\frac{\Delta\varepsilon^2}{32}} + 1/n^2,$$

where the $1/n^2$ term is due to the aforementioned conditioning. Hence, taking the constant c in the definition of Δ to be sufficiently large yields $\Delta \gg \frac{\log n}{\varepsilon^2}$, implying that the total risk is

$$r_{\text{total}} < \frac{\varepsilon}{\sqrt{2}} + O(1/n^2).$$

When n is sufficiently large, this is less than 0.715ε, as required by the theorem.

Next, let us calculate the success probability. For this purpose, we need to lower bound the probability that at least a third of the rebels output "many" when $\rho \geq 0.8$. Without loss of generality, we may assume that $\rho = 0.8$, since for any $\rho > 0.8$, the probability to that u outputs "many" can only increase. Consider a rebel u with degree at least Δ. As before, we condition on the highly likely event that the number of rebels among the neighbors of u is within the interval $[(1-\delta)\rho\Delta_u, (1+\delta)\rho\Delta_u]$. By reasoning similarly to the proof of Claim 2, we can prove that this event happens with probability at least $1-1/n^2$. As before, consider any fixed population of rebel neighbors of u and denote by $\hat{\rho}_u$ the fraction of rebels among the neighbors of u. Taking $\delta = 0.1$ we obtain that $\hat{\rho}_u \in [0.72, 0.88]$.

As before, since we condition on a specific assignment of rebels, each variable s_i is normally distributed. Hence, $\bar{s}$ is also normally distributed according to $N(\mu, \sigma^2)$, where $\mu = \hat{\rho}_u\varepsilon$ and $\sigma^2 = 1/\Delta_u$. By applying the Chernoff bound for a normal distribution (Eq. (1)), the probability that u does not output "many" is

$$P(\bar{s} < \varepsilon/2) \leq P(\mu - \bar{s} \geq 0.22\varepsilon) \leq P\left(|\bar{s} - \mu| \geq \sigma \cdot 0.22\varepsilon\sqrt{\Delta_u}\right) < 2e^{-\frac{(0.22)^2\Delta_u\varepsilon^2}{2}} < 2e^{-\frac{\Delta\varepsilon^2}{50}}.$$

By a union bound argument, the probability that all rebels with degree at least Δ output "many" is at least $1 - 2ne^{-\frac{\Delta\varepsilon^2}{50}} - 1/n^2$, where the $1/n^2$ factor comes from the aforementioned conditioning. Since $\rho = 0.8$, by Claim 1, for n sufficiently large, with high probability (namely, at least $1-1/n^2$), the number of rebels with degree at least Δ is at least $n/3$. By using again a union bound argument, we have that the success probability is at least $1 - 2ne^{-\frac{\Delta\varepsilon^2}{50}} - 2/n^2$. Finally, taking the constant c in the definition of Δ to be sufficiently large (for example, $c > \frac{150}{\varepsilon^2}$) can guarantee that $e^{-\frac{\Delta\varepsilon^2}{50}} \leq 1/n^3$, implying that the success probability is at least $1 - O(1/n^2)$. This concludes the proof of Theorem 1.

D Analysis of the Median protocol

The goal of this section is to prove Theorem 2. As in the proof of Lemma 1, let P_0 (respectively, P_1) denote the normal distribution with mean 0 (respectively, ε) and variance 1. Recall that each obedient citizen sends to any other agent a signal drawn from P_0, while a rebel sends to any other agent a signal drawn from P_1. In the following, we say that a signal is *high* if it is above ε, and, for any two agents u and v, we denote by $\chi_{v\to u}$ the binary random variable such that $\chi_{v\to u} = 1$ if and only if the signal sent by agent v and received from agent u is high. Clearly, we have that, if v is a rebel, then $P(\chi_{v\to u} = 1) = 1/2$, while, if v is an obedient citizen, then $P(\chi_{v\to u} = 1) = \psi(\varepsilon)$, where $\psi(z) = P(x \geq z)$ is the tail distribution of P_0. Let $\overline{\chi_u}$ be the random variable denoting the number of high signals received by an agent u, that is, $\overline{\chi_u} = \sum_{(v,u)\in E(G)} \chi_{v\to u}$. The expected value $E[\overline{\chi_u}]$ of $\overline{\chi_u}$ satisfies the following inequalities:

$$E[\overline{\chi_u}] \geq ((1/2)\rho + \psi(\varepsilon)(1-\rho) - o(1))\,\Delta_u, \tag{5}$$

and

$$E[\overline{\chi_u}] \leq ((1/2)\rho + \psi(\varepsilon)(1-\rho) + o(1))\,\Delta_u, \tag{6}$$

where the term $o(1)$ accounts for the uncertainly that results from the behavior of undercover agents. A good approximation of the cumulative function of the normal distribution[5,6] implies the following upper and lower bounds for the tail distribution:

$$\psi(\varepsilon) \geq \frac{1 - \sqrt{1 - \frac{1}{e^{\sqrt{\frac{\pi}{8}}\varepsilon^2}}}}{2} - \frac{1}{500} > \frac{1}{2}(1-2\varepsilon), \tag{7}$$

and

$$\psi(\varepsilon) \leq \frac{1 - \sqrt{1 - \frac{1}{e^{\sqrt{\frac{\pi}{8}}\varepsilon^2}}}}{2} + \frac{1}{500} < \frac{1}{2}\left(1 - \frac{2}{3}\varepsilon\right), \tag{8}$$

where the second inequalities holds when $\varepsilon \in [0.04, 0.2]$. Equations 5, 6, 7, and 8 imply that

$$E[\overline{\chi_u} \mid \rho \geq 0.8] \geq \left(\frac{2}{5} + \frac{1}{5}\psi(\varepsilon) - o(1)\right)\Delta_u > \left(\frac{1}{2} - \frac{\varepsilon}{5} - o(1)\right)\Delta_u,$$

and that

$$E\left[\overline{\chi_u} \mid \rho \le 0.2\right] \le \left(\frac{1}{10}+\frac{4}{5}\psi(\varepsilon)+o(1)\right)\Delta_u < \left(\frac{1}{2}-\frac{4}{15}\varepsilon+o(1)\right)\Delta_u.$$

We define $\varphi(\varepsilon) = 7\varepsilon/30$ and $f(\varepsilon) = 1/2 - \varphi(\varepsilon)$ (note that $4\varepsilon/15 > \varphi(\varepsilon) > \varepsilon/5$). Let us now calculate the output-risk of a rebel u. This corresponds to the probability of having a false-positive mistake, that is, that u outputs "many" even though $\rho \le 0.2$. Let us therefore condition on having $\rho \le 0.2$. If $\Delta_u < \Delta$ then the rebel u does not output anything and hence there is no output-risk. Hence, aiming to give an upper bound on the output-risk, we may assume without loss of generality that $\Delta_u \ge \Delta$. In this case, agent u outputs "many" if $\overline{\chi_u} > f(\varepsilon)\Delta_u$. We show next that for Δ sufficiently large, this happens with probability at most $e^{-0.015(1-2\varepsilon)\varepsilon^2\Delta}$. Indeed, for any $\rho \le 0.2$, the following holds: $P\left(\overline{\chi_u} > f(\varepsilon)\Delta_u\right) < e^{-0.015(1-2\varepsilon)\varepsilon^2\Delta}$.

Hence, the output-risk is at most $e^{-0.015(1-2\varepsilon)\varepsilon^2\Delta}$. By Lemma 1, the relative message-risk is $r_{\text{message}} \le \varepsilon/\sqrt{2}$, hence, the total risk is

$$r_{\text{total}} = r_{\text{output}} + r_{\text{message}} \le \frac{\varepsilon}{\sqrt{2}} + e^{-0.015(1-2\varepsilon)\varepsilon^2\Delta}.$$

Next, let us calculate the success probability. For this purpose, we need to lower bound the probability that at least a third of the rebels output "many" when $\rho \ge 0.8$. Consider a rebel u with degree at least Δ, i.e., $\Delta_u \ge \Delta$. For Δ sufficiently large, the probability that this rebel does not output "many" given that $\rho \ge 0.8$ is at most $e^{-0.036\varepsilon^2\Delta}$. Indeed, for any $\rho \ge 0.8$, the following holds: $P\left(\overline{\chi_u} < f(\varepsilon)\Delta_u\right) < e^{-0.036\varepsilon^2\Delta}$.

By a union bound argument, the probability that all rebels with degree at least Δ output "many" is at least $1 - ne^{-0.036\varepsilon^2\Delta}$. Since $\rho \ge 0.8$, we have that, by Claim 1, with high probability, the number of rebels with degree at least Δ is at least $n/3$. By using again a union bound argument, we have that the success probability is at least $1 - ne^{-0.036\varepsilon^2\Delta} + 1/n^2$.

Note that for sufficiently large n and assuming $\Delta > \frac{c \log n}{\varepsilon^2}$ for a sufficiently large constant $c > 0$, we get that the success probability is at least $1 - O(1/n^2)$, and the total risk is at most 0.715ε, as stated in the theorem. This completes the proof of Proof of Theorem 2.

E An impossibility result under private communication

The goal of this section is to prove Theorem 3. Consider the private communication framework in a regular network of degree Δ and size n, and let $\mathscr{R}$ be a deterministic uniform rebel protocol. Recall that in such a protocol, each rebel sends the same message m to all its neighbors. Assume that the success probability of $\mathscr{R}$ is at least p, for some constant $p > 0$ and for sufficiently large n and Δ.

We now design a specific police protocol $\mathscr{P}$, called the *Reverse police protocol* that confronts rebels with their own detection protocol. That is, in order to decide whether or not to arrest agent u, the reverse police protocol $\mathscr{P}$ does the following. First, it collects the set $\Lambda_u^{\rightarrow}$ of Δ signals corresponding to the Δ messages outgoing from u. All these signals are samples drawn from the same normal distribution, that is, either $N(0,1)$ or $N(m,1)$ depending on whether u is an obedient citizen or a rebel. Note also that the signal in $\Lambda_u^{\rightarrow}$ viewed by the police, corresponding to the message sent by u to one of its neighbor v is not equal to the corresponding signal received by v; however, the two signals are both samples drawn from the same normal distribution (once again, either $N(0,1)$ or $N(m,1)$). The police protocol $\mathscr{P}$ then simulates, for each agent u, the rebel output decision protocol assuming that the incoming signals of u are the ones in $\Lambda_u^{\rightarrow}$. In other words, $\mathscr{P}$ simulates the scenario in which agent u is a rebel that receives from each of its neighbors v the signal in $\Lambda_u^{\rightarrow}$ corresponding to the message sent by u to v. If the simulation outputs "many" then the reverse police arrests agent u.

In order to analyze the total risk $r_{\text{total}} = r_{\text{output}} + r_{\text{message}}$ of a rebel, we will show that the message-risk of a rebel is at least $p/4$ by analyzing the case in which all agents are rebels (that is, $\rho = 1$), and that the message-risk of an obedient citizen is at most $r_{\text{output}} + 1/n$ by analyzing the case in which almost all agents are obedient (for example, $\rho = 1/(2n^2)$). Since the relative message-risk r_{message} of a rebel is the difference between the message-risk of a rebel and the message-risk of an obedient citizen, we have that $r_{\text{message}} \ge p/4 - r_{\text{output}} - 1/n$. Hence, $r_{\text{total}} \ge r_{\text{output}} + p/4 - r_{\text{output}} - 1/n = p/4 - 1/n$, and the theorem is thus proved. It then remains to prove the two bounds on the message-risk of a rebel and of an obedient citizen.

Bounding the message-risk of a rebel.

Let us consider the case in which all agents are rebels, that is, $\rho = 1$. In this case, by the correctness guarantee, with probability at least p, at least $1/3$ of the agents output "many". Let $\hat{p}_{n,\Delta}$ be the probability that a rebel outputs "many" (assuming a Δ-regular network of size n where all agents are rebels). Hence, the expected fraction of rebels that output "many" is $\hat{p}_{n,\Delta}$. Note that, since $\rho = 1$, each rebel outputs "many" with probability $\hat{p}_{n,\Delta}$ independently of other agents. This is because each

agent (being a rebel) sends the same message m to each of its neighbors. The signals in the system are therefore independent samples taken from $N(m,1)$. The output decision of each rebel is based on its incoming signals which are independent from the incoming signals of other rebels. Because of the Markov inequality, we have that there exists some n_0 such that, for every $n > n_0$, $\hat{p}_{n,\Delta} \geq p/4$. Indeed, let X_u be the random binary variable indicating whether a rebel u outputs "many", and let $X = \sum X_u$. Then $\mu = E[X] = \sum E[X_u] = n\hat{p}_{n,\Delta}$. By contradiction, suppose that $\hat{p}_{n,\Delta} < p/4$. By applying Markov's inequality, we have that

$$P(X \geq n/3) \leq n\hat{p}_{n,\Delta}/(n/3) < 3p/4 < p,$$

contradicting the hypothesis that there exists some n_0 such that, for every $n > n_0$, the success probability is at least p. In what follows we hence assume $n > n_0$.

The decision of rebel u is based on the collection of received signals $\Lambda_u^{\leftarrow}$. As all agents are rebels, as mentioned, all signals follow the same distribution $N(m,1)$. Hence, the signals in $\Lambda_u^{\leftarrow}$ are samples drawn from the same distribution as the signals in $\Lambda_u^{\rightarrow}$ (that is, the set of signals collected by the police protocol). Since a rebel whose input is $\Lambda_u^{\leftarrow}$ outputs "many" with probability at least $p/4$, it also outputs "many" with probability at least $p/4$ when given as input the collection $\Lambda_u^{\rightarrow}$. By the definition of the Reverse police protocol $\mathscr{P}$, it follows that the police arrests a rebel with probability at least $p/4$. In other words, the message-risk of a rebel is at least $p/4$.

Bounding the message-risk of an obedient citizen.

Let us consider the case in which almost all agents are obedient citizens, that is, the case in which ρ tends to zero. More precisely, assume that $\rho = 1/(2n^2)$. In particular, for $n \geq 2$, we have $\rho < 0.2$. The event that there is at least one rebel in the network happens with positive probability. Consider such a rebel r. By the definition of output-risk, based on the set of its incoming signals $\Lambda_r^{\leftarrow}$, rebel r outputs "many" with probability at most r_{output}. Let A be the event that all the Δ neighbors of r are obedient citizens. Then

$$r_{\text{output}} \geq P(r \text{ outputs "many"}) \geq P(r \text{ outputs "many"} \mid A) \cdot P(A).$$

By a union bound argument, the probability that at least one neighbor of r is a rebel is at most $\Delta\rho$. Therefore, $P(A) \geq 1 - \Delta\rho$. Together, we obtain:

$$\frac{r_{\text{output}}}{1-\Delta\rho} \geq P(r \text{ outputs "many"} \mid A). \tag{9}$$

Next observe, that given A, the set of Δ incoming signals $\Lambda_r^{\leftarrow}$ follows the same distribution as $\Lambda_u^{\rightarrow}$, namely, the set of Δ signals outgoing from an obedient citizen u. Hence, by the definition of $\mathscr{P}$, the police arrests an obedient citizen with probability $P(r \text{ outputs "many"} \mid A)$, which is, by Eq. 9, at most

$$\frac{r_{\text{output}}}{1-\Delta\rho} < r_{\text{output}}\left(1+\frac{1}{n}\right) \leq r_{\text{output}} + \frac{1}{n},$$

for sufficiently large n. This completes the proof of Theorem 3.

F Analyzing the Self-Immolation protocol

The goal of this section is to prove Theorem 4. Recall that the theorem assumes that $q = c\log n/\Delta$. A rebel in the self-immolation protocol sends the message 0 with probability $1-q$, and hence, in this case it is indistinguishable from an obedient citizen. Thus, the expected relative message-risk of a rebel is at most q.

Let us next estimate the output-risk of a rebel u. If the degree of u is less than the median degree, i.e., $\Delta_u < \Delta$, then u does not output anything and hence incurs an output-risk of zero. Let us therefore consider the case that $\Delta_u \geq \Delta$. The probability that a neighbor v of u sends the message M is $q\rho$ (that is, the probability that v is a rebel times the probability that v sends the message M). Let X_u be the random variable denoting the number of neighbors of u that send the message M. Hence, the expected value of X_u is $\mu_u = q\rho\Delta_u$. In particular, if $\rho = 0.2$, then $\mu_u = (1/5)q\Delta_u$. Hence, using Chernoff's bound in Eq. (3), the probability that a rebel outputs "many" given that $\rho \leq 0.2$ is

$$P\left(X_u > \frac{c\Delta_u \log n}{2\Delta} \mid \rho \leq 0.2\right) \leq P(X_u > 2\mu_u \mid \rho = 0.2) \leq e^{-\frac{\mu_u}{3}} \leq e^{-\frac{1}{15}q\Delta} = e^{-\frac{c\log n}{15}} = O(1/n^3),$$

for a sufficiently large constant c.

This implies that the output-risk is $O(1/n^3)$, and the expected total risk is $O(q+1/n^3) = O(\log n/\Delta + 1/n^3) = O(\log n/\Delta)$, as stated in the theorem.

Next, let us calculate the success probability. For this purpose, we need to lower bound the probability that at least a third of the rebels output "many" when $\rho \geq 0.8$. Consider a rebel u with degree at least Δ. Using similar arguments to the ones mentioned above, the probability that this rebel does not output "many" given that $\rho \geq 0.8$ is

$$P\left(X_u < \frac{c\Delta_u \log n}{2\Delta} \mid \rho \geq 0.8\right) = O(1/n^3).$$

By a union bound argument, the probability that all rebels with degree at least Δ output "many" is at least $1 - O(1/n^2)$. Since $\rho \geq 0.8$, by Claim 1, we have that, with high probability (namely, at least $1 - O(1/n^2)$), the number of rebels with degree at least Δ is at least $n/3$. By using a union bound argument, we have that the success probability is at least $1 - O(1/n^2)$. This completes the proof of Theorem 4.

References

1. Backstrom, L., Boldi, P., Rosa, M., Ugander, J. & Vigna, S. Four degrees of separation. In *WebSci*, 33–42 (ACM, 2012).
2. Chung, F. & Lun, L. *Complex Graphs and Networks* (American Mathematical Society, 2006).
3. Ramani, A. S., Eikmeier, N. & Gleich, D. F. Coin-flipping, ball-dropping, and grass-hopping for generating random graphs from matrices of edge probabilities. *SIAM Rev.* **61**, 549–595 (2019).
4. Rigollet, P. & Hütter, J.-C. High dimensional statistics (2015).
5. Polya, G. Remarks on computing the probability integral in one and two dimensions. In *Proceeding of the first Berkeley symposium on mathematical statistics and probability*, 63–78 (1945).
6. Aludaat, K. M. & Alodat, M. T. A Note on Approximating the normal distribution function. *Appl. Math. Sci.* **2**, 425–429 (2008).
